# Supplementary material for: Resolving the full spectrum of human genome variation using Linked-Reads
Source: Genome Res. 2019 Apr;29(4):635–45. doi: 10.1101/gr.234443.118 (PMC6442396; doi:10.1101/gr.234443.118)
Supplement: Supplemental Material [file supp_gr.234443.118_Supplemental_longranger-2.2.zip › longranger-2.2.tar.bz2!/lr2.2-public/lib/stan/cmdstan/stan/doc/api/html/operator__unary__plus_8hpp_source.html]

Stan: src/stan/agrad/rev/operators/operator\_unary\_plus.hpp Source File


|  |  |
| --- | --- |
|  | Stan  2.5.0  probability, sampling & optimization |


- Main Page
- Related Pages
- Namespaces
- Classes
- Files

- File List
- File Members

All Classes Namespaces Files Functions Variables Typedefs Enumerations Enumerator Friends Macros Pages

- src
- stan
- agrad
- rev
- operators

operator\_unary\_plus.hpp

Go to the documentation of this file.

1 #ifndef STAN\_\_AGRAD\_\_REV\_\_OPERATORS\_\_OPERATOR\_UNARY\_PLUS\_HPP

2 #define STAN\_\_AGRAD\_\_REV\_\_OPERATORS\_\_OPERATOR\_UNARY\_PLUS\_HPP

3

4 #include <stan/agrad/rev/var.hpp>

5 #include <boost/math/special\_functions/fpclassify.hpp>

6 #include <stan/agrad/rev/internal/precomp\_v\_vari.hpp>

7 #include <stan/math/constants.hpp>

8

9 namespace stan {

10  namespace agrad {

11

43  inline var operator+(const var& a) {

44  if (unlikely(boost::math::isnan(a.vi\_->val\_)))

45  return var(new precomp\_v\_vari(stan::math::NOT\_A\_NUMBER,

46  a.vi\_,

47  stan::math::NOT\_A\_NUMBER));

48  return a;

49  }

50

51  }

52 }

53 #endif

boost::math::isnan

bool isnan(const stan::agrad::var &v)

Checks if the given number is NaN.

**Definition:** boost\_fpclassify.hpp:69

precomp\_v\_vari.hpp

stan::math::NOT\_A\_NUMBER

const double NOT\_A\_NUMBER

(Quiet) not-a-number value.

**Definition:** constants.hpp:53

stan::agrad::vari::val\_

const double val\_

The value of this variable.

**Definition:** vari.hpp:37

stan::agrad::operator+

fvar< T > operator+(const fvar< T > &x1, const fvar< T > &x2)

**Definition:** operator\_addition.hpp:14

stan::agrad::var::vi\_

vari \* vi\_

Pointer to the implementation of this variable.

**Definition:** var.hpp:40

unlikely

#define unlikely(x)

**Definition:** likely.hpp:9

var.hpp

stan::agrad::precomp\_v\_vari

**Definition:** precomp\_v\_vari.hpp:11

stan::agrad::var

Independent (input) and dependent (output) variables for gradients.

**Definition:** var.hpp:27

constants.hpp


---

[ Stan Home Page ]

*© 2011–2014,
Stan Development Team.*
